# Tumor Microenvironment Features as Predictive Biomarkers in Metastatic Differentiated Thyroid Cancer and Their Relationship With 18F-Fluorodeoxyglucose Positron Emission Tomography/Computed Tomography (18F-FDG PET/CT) Metabolic Parameters

**DOI:** 10.7759/cureus.44751

**Published:** 2023-09-05

**Authors:** Selin Soyluoglu, Ebru Tastekin, Burak Andac, Ulku Korkmaz, Seyma Gizem Orun, Gulay Durmus Altun

**Affiliations:** 1 Nuclear Medicine, Trakya University, Faculty of Medicine, Edirne, TUR; 2 Pathology, Trakya University, Faculty of Medicine, Edirne, TUR; 3 Endocrinology, Diabetes and Metabolism, Trakya University, Faculty of Medicine, Edirne, TUR

**Keywords:** tumor microenvironment, prognosis, fibrosis, lymphocytosis, pet/ct, thyroid cancer

## Abstract

Objective: The role of the tumor microenvironment in tumor progression and treatment response is being investigated for different types of cancer. This study aimed to determine the relationships between tumor microenvironment, histopathology, 18F-fluorodeoxyglucose positron emission tomography/computed tomography (18F-FDG PET/CT)-based metabolic parameters, treatment response, and overall survival (OS) in metastatic differentiated thyroid cancer (DTC).

Methods: Metastatic DTC patients who underwent 18F-FDG PET/CT between 2015-2019 were evaluated. Clinicopathological, histopathological features and PET/CT parameters of patients were recorded. Microenvironmental characteristics of the primary tumor, such as mitosis, intratumoral and peritumoral lymphocytosis, intratumoral and peritumoral fibrosis, were evaluated from the tissue samples. The relationships between these factors were statistically analyzed.

Results: Sixty-five patients (38 females, 27 males, age: 49±15 years) were included. Mitosis, intra/peritumoral lymphocytosis, and intra/peritumoral fibrosis were frequent; however, none of them had a statistically significant association with PET-positive metastases, treatment response, or OS. Univariate analysis showed that gender, size, thyroglobulin values, residual thyroid tissue, PET-positive metastases, and maximum standardized uptake value (SUVmax) were significant predictors of OS. At multivariate analysis, PET-positive metastases (HR=-2.65, 95%CI 0.007−0.707, p=0.024) and SUVmax (HR=-2.74, 95%CI 0.006−0.687, p=0.023) were the only independent predictors for OS.

Conclusion: Our study revealed that microenvironmental characteristics of the primary tumor did not show prognostic significance in metastatic DTC. PET-positive metastases and SUVmax levels were the only significant factors that predicted overall survival in DTC. Supporting the results of our study with further studies with a larger sample size may be necessary to determine the relationship between the tumor microenvironment and prognosis in DTC.

## Introduction

Differentiated thyroid cancers (DTC) are the most common malignancy of the endocrine system [1]. Although they mostly have a favorable prognosis, approximately 10-30% present with metastasis or recurrent disease. The clinical course of metastatic DTC is quite variable.

Patients’ routine follow-ups include serum thyroglobulin (Tg) measurement, cervical ultrasound, and radioactive iodine (RAI or ^131^I) whole-body scan (WBS). 18F-fluorodeoxyglucose positron emission tomography/computed tomography (18F-FDG PET/CT) is used in DTC, especially in patients with poorly differentiated tumors with elevated Tg and negative WBS. Studies have shown that 18F-FDG PET/CT can cause up to 50% changes in clinical management of DTC [2]. Metastatic lesions that do not show ^131^I accumulation but show a high FDG uptake are associated with more aggressive disease [3]. Few studies have investigated the relationship between histopathological features of the primary tumor and FDG uptake in PET/CT for thyroid cancer [3,4]. 

The tumor microenvironment consists of cancer-associated fibroblasts and immune cells and is considered to have an important role in tumor progression and response to treatment. Therefore it is being researched for different types of cancer, including thyroid carcinoma [5]. These cells are involved in tumor progression through cell proliferation, extracellular matrix remodeling, angiogenesis, epithelial mesenchymal transformation, and immune suppression. Studies to date have reported that suppression of cancer-associated fibroblasts can be targeted as a treatment alternative, especially in refractory thyroid cancers. Immune cells, another component of the microenvironment, are also may be possible treatment targets. Studies on the tumor microenvironment in thyroid cancers are still scarce and mostly preclinical, so further studies are warranted [6].

This study aims to investigate tumor microenvironment features such as mitosis, intratumoral/peritumoral lymphocytosis, and intratumoral/peritumoral fibrosis in metastatic papillary thyroid cancer (PTC) and to determine the relationships between these microenvironment features, tumor histopathology, 18F-FDG PET/CT-based metabolic findings, treatment response, and overall survival (OS). The tumor microenvironment is important in terms of personalized treatment approaches, especially in treating thyroid cancers where other treatments are insufficient, so imaging methods will become increasingly important in the selection, treatment planning, and evaluation of treatment efficacy in these patients. To our best knowledge, this is the first study to investigate tumor microenvironment features with the metabolic parameters in 18F-FDG PET/CT together in PTC.

## Materials and methods

Patients

PTC patients referred to PET/CT between January 2015-December 2019 with indication of elevated serum Tg and thyroglobulin antibody (TgAb) levels, or suspicious radiological lesions that could not be clarified by ^131^I WBS, were retrospectively evaluated. Patients whose pathology samples and necessary clinicopathological data were available were included in the study. Patients who were lost to follow-up or died due to non-thyroid causes were excluded. Gender, age at the time of diagnosis, TNM staging, American Thyroid Association (ATA) risk stratification, treatments, initial (post-operative sixth-eighth week), and final (last follow-up) serum Tg and TgAb levels under thyroid stimulating hormone (TSH) stimulation were recorded. All patients had a total thyroidectomy, and prophylactic central lymph node (LN) dissection was performed in 36 patients. Tumor size, extrathyroidal extension, surgical margin, multifocality, intraglandular dissemination, variants, and vascular/lymphovascular/perineural invasions were recorded.

Microenvironment

Microenvironmental features mitoses, lymphocytosis, and fibrosis were evaluated by an expert pathologist. Mitosis count was evaluated in a 2mm^2^ area of a light microscope (Eclipse E600; Nikon, Tokyo, Japan). Fibrosis was evaluated separately for tumor tissue (intratumoral) and stromal tissue surrounding the tumor (peritumoral) and graded as 0: absent; 1: minimal (occupying <50% of the area); 2: moderate (occupying 50-90% of the area); 3: extensive (occupying >90% of the area) on hematoxylin and eosin (H&E) staining [7]. Considering the stromal reaction caused by preoperative fine needle biopsy, sections taken from the other side of the lesion were used for scoring. Lymphocytes were evaluated at higher magnification separately for intratumoral and peritumoral areas. The percentage of lymphocytes was evaluated and scored as grade 0: no inflammation, 1: 1-10%, 2: 10-40%, 3: 40-90% on H&E [8]. 

Imaging and image analysis 

After TSH stimulation (TSH>30 mIU/L), achieved with discontinuation of thyroid hormone and iodine-poor diet for four to six weeks, the patients received a cumulative dose of 275±199 mCi (minimum: 65, maximum: 900 mCi) ^131^I therapy per patient. WBS was performed three to five days after administering ^131^I using a SPECT/CT scanner (Optima NM/CT 640; GE Medical Systems, Milwaukee, WI, USA). The scanner was equipped with high-energy parallel-hole collimators, peaked at 364 keV, 20% window. Anterior/posterior whole-body images were obtained at 8 cm/min speed, 512x512 matrix. Spot anterior/posterior views of the neck were routinely included. Single-photon emission CT (SPECT)/CT was performed to provide more precise localization in patients with suspicious ^131^I accumulation. Additional treatment doses ranging from 100-200 mCi were administered every six to 12 months in 24 patients with ^131^I avid metastases.

Patients were referred to PET/CT with elevated Tg, TgAb, or suspicious radiological lesions that could not be clarified by ^131^I WBS. Discontinuation of thyroid hormone was not applied for PET imaging, as previous studies reported that serum TSH levels do not significantly affect FDG-PET results [9]. After fasting for at least four hours, patients with blood glucose <200 mg/dl received intravenous injection of approximately 3.7 MBq/kg of FDG. An FDG-PET scan was performed one hour after injection using a combined PET/CT system (Discovery STE; GE Medical Systems). CT images were taken from the skull to the mid-thigh by adjusting the CT parameters to 120 kV, 200 mAs with 16 slice CT covering 20 mm, with a thinner slice thickness of 1.25 mm. PET images were recorded in 3D mode, a matrix size of 256×256, four min per bed position, and reconstructed by an iterative method, after CT scan. Axial, coronal, and sagittal reformatted images and PET-CT fusion images were created. 

WBS and PET/CT scans were performed within one year in 86.2% of the patients and within an average of four years (two to six years) in the remaining 13.8%. PET/CT was post-operative in all patients, post-RAI in 28 patients, and pre-RAI in 37 patients. Patients who underwent pre-RAI PET/CT imaging were in the high-risk group for post-operative distant metastasis. Therefore, PET/CT imaging was added to staging for distant metastasis and prognostic determination without waiting for RAI. PET/CT imaging was performed at least four weeks after surgery and RAI treatment to avoid misinterpretations due to inflammation. The first PET/CT performed in patients with multiple PET scans was considered as the reference [3].

WBS and PET/CT images were interpreted blindly by two nuclear medicine physicians. The foci of pathological uptake were examined. A consensus was obtained to classify lesions as metastatic by examining follow-up PET, CT, USG images, histopathological sampling findings, Tg measurements, and treatment response. The maximum standardized uptake (SUVmax) values were obtained for metastatic lesions. 

Statistical analysis

Statistical analyses were performed using SPSS version 25 (IBM Corp., Armonk, NY, USA). The relationship between the microenvironment, histopathological findings, gender, metabolic findings, and treatment response was evaluated by Chi-square and Fisher’s exact test. Student’s t-tests were used for the analysis of paired independent samples. P<0.05 was considered significant. Univariate analyzes were conducted to analyze the effect of age, gender, TNM staging, ATA risk stratification, Tg values, microenvironmental and histopathological features, WBS-positive/PET-positive metastases, and SUVmax on survival. Multivariate analyses were performed using the Cox proportional hazard model to identify the best independent factors. Only variables that predicted OS by univariate analyses were included in the multivariate analysis. Survival curves were created using Kaplan-Meier estimates. Receiver operating characteristic (ROC) curves were created to determine a SUVmax cut-off value, providing optimal sensitivity and specificity to predict survival. Patients were divided into two groups according to SUVmax cut-off values, and Kaplan-Meier analysis was performed.

## Results

Patient characteristics

Sixty-five PTC patients (38 females, 27 males) were included in the study. Only one pediatric patient, 11 years old, was included in the study. The mean age at diagnosis was 48±15 (11-75) years. Six patients (9%) had micro PTCs. The mean tumor size was 2.4±1.6 (0.2-8) cm. Tumor histological variants, ATA risk stratifications, T and N stages, and the other details of patient characteristics and the relationship of these factors to treatment response and OS are given in Table 1.

**Table 1:** Detailed patient characteristics and their relationship to treatment response and death Inv: invasion, Ext: extension, ITL: intratumoral lymphocytosis, PTL: peritumoral lymphocytosis, ITF: intratumoral fibrosis, PTF: peritumoral fibrosis, NS: not significant *Statistically significant **Complete response: Evaluated in available 59 patients

	Number of patients	Complete response** (%within group)	p	Deaths (%)	p
All patients	65	31 (52.5)		7 (10.8)	
Age			NS		NS
<55 yr	43	21 (55.3)		4 (9.3)	
≥55 yr	22	10 (47.6)		3 (13.6)	
Gender			NS		0.026*
Female	38	19 (52.8)		2 (5.3)	
Male	27	12 (52.2)		5 (18.5)	
Size			0.011*		0.020*
<1 cm	7	2 (42.9)		0 (0)	
1-4 cm	45	26 (65.0)		3 (6.7)	
≥4 cm	13	2 (16.7)		4 (30.8)	
Variants			NS		NS
Classical	28	13 (52)		2 (7.1)	
Follicular	18	10 (58.8)		1 (5.6)	
Hurtle cell	6	3 (60)		0 (0)	
Tall cell	5	3 (60)		0 (0)	
Columnar	2	1 (100)		1 (50)	
Clear cell	2	0 (0)		1 (50)	
Solid	2	1 (50)		0 (0)	
Poorly diff	2	0 (0)		2 (100)	
T stage			NS		NS
T1a	5	3 (60)		0 (0)	
T1b	13	7 (63.6)		1 (7.7)	
T2	7	4 (80)		1 (14.3)	
T3a	9	5 (62.5)		0 (0)	
T3b	26	11 (44)		3 (11.5)	
T4a	5	1 (20)		2 (40)	
N stage			NS		NS
N0	17	11 (68.8)		2 (11.8)	
N1a	7	4 (57.1)		0 (0)	
N1b	12	4 (33.3)		0 (0)	
Nx	29	12 (50)		5 (17.2)	
Blood vessel inv.			NS		NS
Presence	1	0 (0)		1 (100)	
Absence	64	31 (53.4)		6 (9.4)	
Lymphovascular inv.			0.007*		NS
Presence	25	8 (32)		3 (12)	
Absence	40	23 (67.6)		4 (10)	
Extrathyroidal ext.			NS		NS
Absence	27	16 (66.7)		2 (7.4)	
Minimal	7	4 (57.1)		1 (14.3)	
Gross	31	11 (39.3)		4 (12.9)	
Multifocality			NS		NS
Unifocal	29	13 (50)		3 (10.3)	
Multifocal	36	18 (54.5)		4 (11.1)	
Surgical Margin			0.013*		NS
Positive	42	16 (41)		5 (11.9)	
Negative	23	15 (75)		2 (8.7)	
Capsule inv.			NS		NS
Presence	40	19 (50)		2 (5)	
Absence	25	12 (57.1)		5 (20)	
Perineural inv.			NS		NS
Presence	5	1 (20)		1 (20)	
Absence	60	30 (55.6)		6 (10)	
Intraglandular dissemination			NS		NS
Presence	7	2 (28.6)		2 (28.6)	
Absence	58	29 (55.8)		5 (8.6)	
ATA stratification			0.006*		NS
Low	7	7 (100)		0 (0)	
Intermediate	9	6 (75)		1 (11.1)	
High	49	18 (40.9)		6 (12.2)	
Mitosis			NS		NS
0	2	2 (100)		0 (0)	
1	34	14 (48.3)		5 (14.7)	
2	21	13 (65.0)		1 (4.8)	
3	7	2 (28.6)		1 (14.3)	
4	1	1 (100)		0 (0)	
ITL			NS		NS
0	34	17 (51.5)		3 (8.8)	
1	22	9 (52.9)		3 (16.3)	
2	8	4 (50)		1 (12.5)	
3	1	1 (100)		0 (0)	
PTL			NS		NS
0	28	13 (48.1)		3 (10.7)	
1	17	11 (73.3)		0 (0)	
2	9	2 (28.6)		1 (11.1)	
3	11	5 (50)		3 (27.3)	
ITF			NS		NS
0	18	12 (75)		2 (11.1)	
1	12	5 (50)		1 (8.3)	
2	16	6 (40)		2 (12.5)	
3	19	8 (44.4)		2 (10.5)	
PTF			NS		NS
0	8	6 (75)		1 (12.5)	
1	22	9 (47.4)		2 (9.1)	
2	25	11 (50)		2 (8)	
3	10	5 (50)		2 (20)	

Multifocality, positive surgical margin, capsule invasion, lymphovascular invasion, and extrathyroidal extension (55.4%, 64.6%, 62.5%, 38.5%, 58.5%, respectively) were frequently detected, while blood vessel invasion, perineural invasion, and intraglandular dissemination were rare (1.5%, 7.7%, 10.8%, respectively). 

Of 36 patients who underwent prophylactic central lymph node dissection during thyroidectomy, 17 patients were staged as N0, eight patients as N1a and 11 patients as N1b.

Microenvironment features

Mitosis was observed in 96.9% of the patients. Mitosis count in a 2mm^2^ area was one in 34, two in 21, three in seven, and four in one patient. 

Intratumoral lymphocytosis (ITL) was observed in 47.7% of the patients (22 grade 1, eight grade 2, one grade 3). Peritumoral lymphocytosis (PTL) was observed in 56.9% of the patients (17 grade 1, nine grade 2, 11 grade 3). 80.6% of patients with ITL were under the age of 55 (p=0.018). 

Intratumoral fibrosis (ITF) was observed in 72.3% of the patients (12 grade 1, 16 grade 2, 19 grade 3). A relationship was observed between ITF and lymphovascular invasion, extrathyroidal extension, positive surgical margin, and multifocality (p=0.025, 0.011, 0.001, 0.025, respectively). Patients with ITF were mostly in the ATA high-risk group (p=0.013). Peritumoral fibrosis (PTF) was observed in 87.7% of the patients (22 grade 1, 25 grade 2, 10 grade 3). A significant relationship was found between PTF and extrathyroidal extension, positive surgical margin (p=0.040, 0.019, respectively). 

A correlation was found between ITL and PTL (r=0.641, p<0.001); ITF and PTF (r=0.402, p=0.001); and PTL and PTF (r=0.467, p<0.001) grades. 

PET/CT and WBS findings 

In total, 45 (69.2%) patients had PET-positive lesions, and 18 (27.7%) had WBS-positive lesions. While 27 (41.5%) patients were only PET-positive, 18 (27.7%) patients were both PET-positive and WBS-positive. No lesion was detected on either scan in 20 (30.8%) patients. The patients' treatment response and survival status according to PET/CT and WBS findings are given in Table 2.

**Table 2:** Patients based on PET/CT and WBS results PET/CT: positron emission tomography/computed tomography, WBS: Control whole body scan after one year of initial ^131^I treatment * Statistically significant **Complete response: Evaluated in available 59 patients

	Total Number of patients	Complete Response** (%within group)	p	Deaths	p
PET/CT			0.001*		0.037*
Positive	45	13 (33.3)		7 (10.8)	
Negative	20	18 (90)		0 (0)	
I-131 WBS			0.002*		0.287
Positive	18	3 (18.8)		3 (4.6)	
Negative	47	28 (65.1)		4 (6.2)	

Forty-one (63.1%) patients had PET-positive LN, with an average SUVmax of 5.4±5.0 (1.1-28.3). Nine (13.9%) patients had PET-positive bone lesions, with an average SUVmax of 6.9±2.8 (4-12.6). Three (4.6%) patients had PET-positive liver lesions, with an average SUVmax of 6.9±2.0 (4.6-8.1). No isolated liver metastases were detected. Thirteen (20%) patients had PET-positive lung metastases. Three of them were micronodular (miliary, diffusely reticular pattern), and 10 of them were macronodular (distinct nodules visible on conventional radiology), with an average SUVmax of 5.2±5.1 (0.7-13.7 

**Figure 1:**
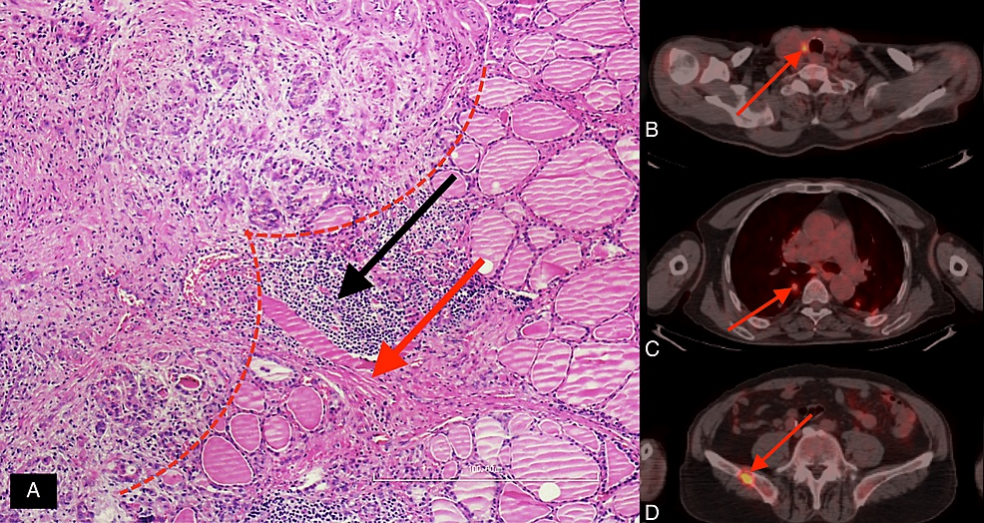
Pathology and PET/CT findings of a patient A 70-year-old male with no intratumoral but peritumoral grade 3 lymphocytosis (A, black arrow) and fibrosis (A, red arrow). PET/CT showed cervical lymph node (B, arrow), lung (C, arrow), and bone metastases (D, arrow). The patient died of DTC one year after diagnosis. PET/CT: positron emission tomography/computed tomography, DTC: differentiated thyroid cancer

**Figure 2:**
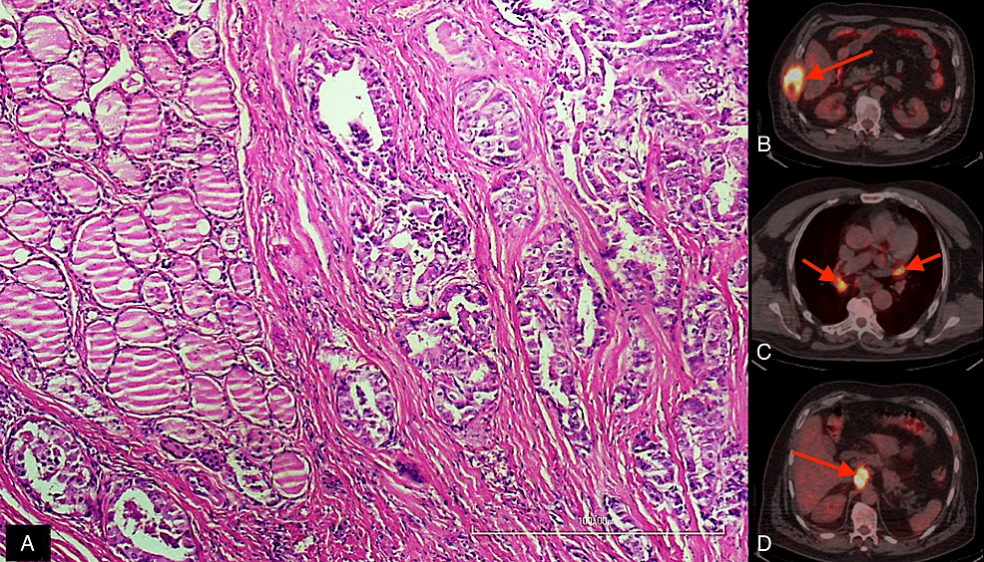
Pathology and PET/CT findings of an another patient A 52-year-old male with moderate intratumoral and peritumoral fibrosis without intratumoral or peritumoral lymphocytosis (A). PET/CT showed bone (B, arrow), mediastinal lymph node (C, arrows), and abdominal lymph node metastases (D, arrow). The patient died of DTC seven years after diagnosis. PET/CT: positron emission tomography/computed tomography, DTC: differentiated thyroid cancer

No significant relationship between PET/CT findings and the mitosis or intra/peritumoral lymphocytosis and fibrosis was found (Figures 1, 2).

The mean SUVmax was 4.7±5.1. There was a weak positive correlation between the post-operative Tg (r=0.443, p=0.001), final Tg (r=0.312, p=0.012), the first RAI dose (r=0.289, p=0.020), the cumulative RAI dose (r=0.366, p=0.006) and SUVmax (Table 3). 

**Table 3:** Correlation of risk factors with SUVmax Tg: thyroglobulin (ng/ml), RAI: radioactive iodine (mCi), SUVmax: maximum standardized uptake value * Statistically significant

	Mean	Correlation with SUVmax	
		r	p
SUVmax	6.6 ± 5.0		
Age at diagnosis	48 ± 15	0.213	0.088
Tumor size (cm)	2.4 ± 1.6	0.110	0.385
Initial Tg	56.3 ± 94.8	0.443	0.001*
Final Tg	37.6 ± 93.5	0.312	0.012*
First RAI dose	147.8 ± 40	0.289	0.020*
Cumulative RAI dose	275.5 ± 199.2	0.366	0.006*

Radioiodine treatment and treatment response

All patients received radioiodine treatment at least one and at most six times. The mean first RAI dose was 147.8 mCi, and the cumulative dose was 275.5 mCi (65 mCi - 1150 mCi). The cumulative dose received by the patients was correlated with initial Tg (r=0.525, p<0.001) and TgAb (r=0.269, p=0.049). Patients without biochemical (stimulated Tg <2ng/ml) or structural evidence of disease at the last follow-up were considered 'complete responders'. Thirty-one patients had a complete response. Factors associated with complete response are also in Table 1. 

Overall survival

All patients were followed up for a mean period of 11.3±0.7 years (range 1-13 years). Seven patients died at a mean of 3±2.0 (median: 3) years during follow-up. The median age at death was 52 years. 

The overall two-year survival rate was 98.5%, and the five-year survival rate was 92.3%. All seven patients who died had PET-positive metastatic lesions, and when analyzed separately, five-year survival was 84% in PET-positive patients and 100% in PET-negative patients. 

**Figure 3:**
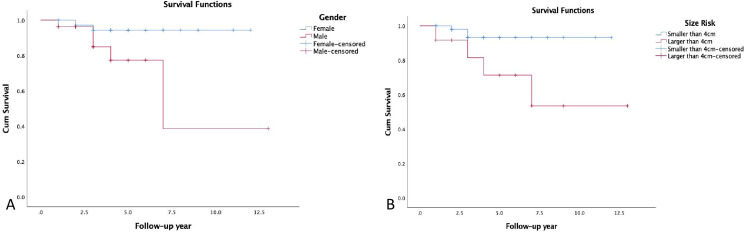
Kaplan Meier curves for overall survival based on gender (A), size (B).

**Figure 4:**
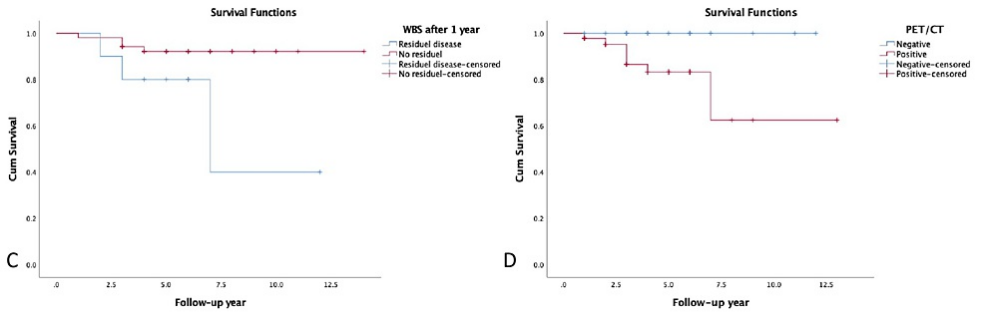
Kaplan Meier curves for overall survival based on residual disease after RAI treatment (C), FDG avid distant metastases (D). PET/CT: positron emission tomography/computed tomography, WBS: whole body scan, RAI: radioactive iodine

**Figure 5:**
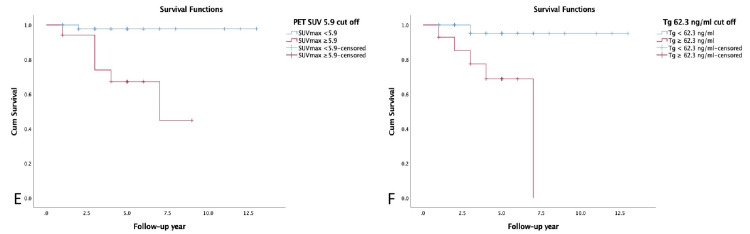
Kaplan Meier curves for overall survival based on SUVmax values (E), and Tg values (F). PET: positron emission tomography, SUVmax: maximum standardized uptake value, Tg: thyroglobulin

In univariate analysis, OS was related to gender (p=0.026), tumor size (p=0.020), Tg values (p=0.006), residual thyroid tissue (p=0.038), PET-positive metastases (p=0.037), and SUVmax (p=0.001) (Figures 3-5). The analysis revealed a SUVmax cut-off value of 5.9 (area under the ROC curve (AUC)=0.887, SE=0.042; 95%CI=0.805-0.968; sensitivity 85.7%, specificity 81.0%) and a Tg cut-off value of 62.3 ng/ml (AUC=0.716, SE=0.136; 95%CI=0.448-0.983; sensitivity 71.4%, specificity 84.5%) to predict OS. 

Except for tumor size, neither the classical histopathological findings nor the microenvironmental parameters were associated with survival. 

The following variables were included in the multivariate analysis: gender, size, initial Tg, PET-positive metastases, SUVmax, and residual post-RAI remnant. The presence of PET-positive metastases (HR=-2.65, 95%CI 0.007−0.707, p=0.024) and SUVmax (HR=-2.74, 95%CI 0.006−0.687, p=0.023) were the only two independent significant predictors.

## Discussion

PTC generally has a favorable prognosis, and most patients have a good chance of getting an excellent response. It is a cause for concern that patients with PTC may be exposed to overtreatment complications. Therefore, the necessity and dose reduction of radioiodine ablation therapy is still a matter of debate. However, the disease can metastasize and cause death in a small number of patients. These patients are mostly resistant to radioactive iodine treatment, and alternative treatment methods, such as tyrosine kinase inhibitors, are applied. Thus, biomarkers that can guide the risk assessment of treatment resistance, novel patient-specific treatment options, and prognostic prediction are gaining importance.

Classical histopathological data were found to be insufficient in this regard, and therefore, studies continue on new molecular, genetic, and microenvironmental biomarkers that can predict tumor progression and survival. Due to the low incidence of metastatic DTC, there are few studies on the molecular, genetic, and microenvironmental characteristics of thyroid carcinoma patients with distant metastases. Although more studies with more patients are needed, current research has promising findings [10,11]. Additionally, there are studies investigating the potential value of the combination of ^124^I PET/CT and 18F-FDG PET/CT in preventing unnecessary ^131^I treatments in patients with suspected recurrence [12]. It seems that new studies involving molecular imaging are needed to select patients with poor prognoses and decide on personalized treatment options that may benefit these patients. In the present study, we demonstrated that cancer-related fibrosis and lymphocytosis detected in the peritumoral or intratumoral area do not provide any helpful insight regarding prognosis or metabolic tumor behavior. Moreover, the presence of 18F-FDG PET-positive metastases and SUVmax values at follow-up were the only independent significant predictors for the overall survival of PTC. However, further clinical trials with higher sample sizes are necessary to establish solid evidence of the absence of an association between PTC and the tumor microenvironment.

The tumor microenvironment has been shown to play an important role in tumor aggressiveness and response to therapy in cancer. A better prognosis has been reported in the presence of lymphocytic infiltrates for various cancers [13]. The prevalence of chronic lymphocytic thyroiditis (CLT) in PTC patients ranges from 0.5% to 38% [14-16]. For DTC with lymphocytic thyroiditis, lower recurrence and mortality rates have been reported [16-19]. Various theories have been proposed to explain how the coexistence of CLT leads to a better prognosis in PTC. One study found that follicular cells in the CLT activate the FAS-mediated apoptosis pathway by expressing both FAS and the FAS ligand [20]. This pathway was thought to cause the death of both normal thyroid tissue and cancer cells. Furthermore, it is worth considering that the immune response directed against the tumor in CLT may lead to less aggressive tumor behavior. Additionally, Kimura et al. [21] stated that the secretion of interleukin-1 by lymphocytes inhibits the growth of thyroid cancer cells. However, contrary to these studies, it has also been claimed that the coexistence of CLT does not have a protective effect on patient outcomes. In a previous study, DTC patients were classified into three groups according to lymphocyte infiltration as diffuse, peritumoral, and absent; the groups showed no differences in regional, nodal, or distant metastases [22]. Another study reported that the presence of lymphocytic thyroiditis was a risk factor for DTC; however, it was not related to extrathyroidal extension or nodal metastasis [23]. Conflicting results regarding the effect of CLT on PTC outcomes may be related to subtypes of lymphocytes in the background of thyroiditis, the definition of CLT, or the age and ethnicity differences of study populations [15,16,24]. To our best knowledge, this is the first study to investigate intratumoral/peritumoral lymphocytosis and fibrosis with the metabolic findings together in DTC. In the present study, the presence or the degree of intratumoral or peritumoral lymphocyte infiltration showed no difference in PET-positive metastases, treatment response, or OS. We found that younger age was the only factor significantly associated with ITL. This finding supported previous studies [25]. In addition, we found that the grade of intratumoral and peritumoral lymphocytosis were correlated. 

Interstitial fibrosis is characterized by the accumulation of fibroblasts and collagen fibers. In previous studies, the effect of fibrosis in the stromal tissue surrounding the tumor [26] or within the tumor [27] was investigated for various cancers and reported to be associated with an increased recurrence rate and decreased OS. Few studies investigated the relationship between fibrosis and DTC [28,29]. Cancer-related fibrosis has been shown to be a poor prognostic factor and is suggested to be an important diagnostic tool for papillary microcarcinoma [28]. Our intratumoral and peritumoral fibrosis rates were higher than in the literature [29,30]. Contrary to previous studies [29,30], our results showed that intratumoral and peritumoral fibrosis grades were correlated. Extrathyroidal extension and positive surgical margin were found to be associated with the presence of intratumoral and peritumoral fibrosis. Patients with intratumoral fibrosis were mostly in the ATA high-risk group, and an association with lymphovascular invasion was observed. However, there was no significant association between 18F-FDG PET-positive metastasis, OS, and intratumor/peritumoral fibrosis. 18F-FDG PET/CT is not a method that can specifically show cancer-related fibroblast activity. However, PET/CT imaging labeled with fibroblast activation protein inhibitor (FAPI PET/CT), which is increasingly used for many types of cancer, including thyroid cancer [31]. It is especially important in terms of promising an advanced treatment option in iodine-negative patients [32]. However, 18F-FDG PET/CT is still the more widely used method due to its high image resolution and easier accessibility in most centers. Future studies will demonstrate the importance of cancer-associated fibroblasts and the role of FAPI PET/CT in the management of thyroid cancer.

Higher necrosis and mitosis rates are primarily seen in poorly differentiated cancer but also in papillary and follicular carcinomas. Undifferentiated cancers had significantly higher mitotic rates than papillary, follicular, and medullary cancers, but the differences in mitotic rates between the last three types were also significant. Previous studies have considered that the presence of mitoses might be useful in predicting prognosis in endocrine cancers. Higher mitosis rates indicated lower differentiation, poor prognosis, and higher mortality rates for thyroid cancer [33]. In our study, it was remarkable that mitosis was observed in most of our patients (96.9%). However, these high mitosis rates are to be expected since all patients were already referred to PET/CT with poor outcomes and high suspicion of metastasis.

Preoperative PET/CT in DTC management is not an effective method considering the cost and radiation burden. However, studies have shown that PET-positive metastases and SUVmax are the only independent prognostic factors for OS in DTC [3,34]. We found that OS was related to the male gender, tumor size, Tg values, residual thyroid tissue, PET-positive metastases, and SUVmax values in the univariate analysis. Gender disparity in incidence, aggressiveness, and prognosis of thyroid cancer is well established, but the reason for the disparity is not fully understood. Some studies have shown that thyroid cancer is more prevalent in women. However, male patients tend to have higher mortality rates from thyroid cancer than females [35,36]. Like previous studies, the multivariate analysis showed that PET-positive metastases and SUVmax were the only independent predictive factors for survival. A previous study reported a 60% two-year OS rate for patients with PET-positive lesions and a 100% rate for patients with negative PET/CT [3]. They reported that survival was similar in PET-positive patients regardless of radioiodine uptake. Similarly, we found an 84% five-year OS for patients with PET-positive lesions, while 100% five-year OS for PET-negative patients. 

Investigating the prognostic impact of a wide variety of factors and their relationship to PET/CT, Deandreis et al. [3] reported that age and necrosis were independent predictive factors of FDG uptake. Some studies showed an association between FDG uptake, tumor size [37,38], and BRAF V600E mutation [37]. The combination of PET/CT findings and Tg levels has been reported to be crucial in the patient management of DTC [39]. We found a positive correlation between initial Tg, final Tg, and SUVmax. The frequency of PET-positive metastases was higher in patients with a higher ATA risk group. These findings supported previous studies [38]. 

As limitations of the study, a histopathological correlation could not be made for all lesions considered metastatic. However, additional imaging methods, repeated PET scans, and sufficient follow-up periods have helped characterize the lesions. Secondly, patients with PTC often have an excellent prognosis and long life expectancy, so patient numbers were low for some subgroups in survival analyses. In addition, all our patients were those who already applied to PET/CT with a high suspicion of metastasis. Further studies with larger numbers of patients are warranted to confirm our findings and determine the cutoff points for an accurate prediction.

## Conclusions

Despite a generally favorable prognosis for DTC, some patients may develop metastases and require alternative treatments. Microenvironmental biomarkers and imaging methods are being studied to personalize treatment and follow-up better. Traditional histopathological data is insufficient for predicting tumor progression and survival, so new microenvironmental biomarkers are being explored. In our study investigating the prognostic importance of the tumor microenvironment in PTC together with 18F-FDG PET/CT-based metabolic parameters, we demonstrated that microenvironmental characteristics of the primary tumor, such as fibrosis and lymphocytosis detected in the intratumoral or peritumoral area, did not show prognostic significance in metastatic PTC. In addition, PET-positive metastases and SUVmax values of lesions at follow-up were identified as the only independent significant predictive factors for OS of PTC. Our research results may be significant as they provide clinical evidence of no association between tumor microenvironment characteristics and PTC prognosis. However, further clinical trials with higher sample sizes are necessary to establish solid evidence of the absence of an association between PTC and the tumor microenvironment.
